# Adsorptive Removal of Anionic Azo Dye New Coccine Using Silica and Silica-gel with Surface Modification by Polycation

**DOI:** 10.3390/polym13101536

**Published:** 2021-05-11

**Authors:** Tien Duc Pham, Viet Phuong Bui, Thuy Nga Pham, Thi Mai Dung Le, Kim Thuy Nguyen, Van Hoi Bui, The Dung Nguyen

**Affiliations:** 1 Faculty of Chemistry, University of Science, Vietnam National University—Hanoi, 19 Le Thanh Tong, Hoan Kiem, Hanoi 100000, Vietnam; buivietphuong_t63@hus.edu.vn (V.P.B.); phamthuynga_sdh@hus.edu.vn (T.N.P.); 2 Faculty of Environmental Sciences, University of Science, Vietnam National University—Hanoi, 334 Nguyen Trai, Thanh Xuan, Hanoi 100000, Vietnam; lethimaidung_t63@hus.edu.vn; 3 Vietnam-Russia Tropical Centre, 63 Nguyen Van Huyen, Cau Giay, Hanoi 100000, Vietnam; nguyenkimthuy@vrtc.org.vn; 4 Vietnam Academy of Science and Technology, University of Science and Technology of Hanoi (USTH), 18 Hoang Quoc Viet, Cau Giay, Hanoi 100000, Vietnam; bui-van.hoi@usth.edu.vn

**Keywords:** new coccine, adsorption, silica, silica-gel, PDADMAC

## Abstract

In the present work, adsorption of anionic azo dye, new coccine (NCC) on silica and silica-gel in an aquatic environment was discovered. Effective conditions such as adsorption time, pH, the influence of dosage on NCC adsorption using strong polycation, poly-diallyl-dimethylammonium chloride (PDADMAC) modified silica (PMS) and PDADMAC modified silica-gel (PMSG) were systematically studied. The removal of NCC using PMS and PMSG were much higher than that using raw silica and silica-gel without PDADMAC in all pH ranges from 3 to 10. The adsorption of NCC onto PMS and PMSG was achieved maxima at the same conditions of contact time 30 min, pH 6. The optimum adsorbent dosages of PMS and PMSG for NCC removal were 10 and 20 mg·mL^−1^, respectively. Experimental results of NCC adsorption isotherms onto PMS and PMSG at different ionic strength were fitted by Langmuir and Freundlich models. The NCC removal efficiencies using PMS and PMSG were higher than 87%, indicating that PMS and PMSG are novel and reusable adsorbents for removal of anionic dye. Based on adsorption isotherms, and surface group changes after PDADMAC modification and NCC adsorption examined by Fourier transform infrared spectroscopy (FTIR), we demonstrate that electrostatic interaction between positively charged adsorbents’ surfaces and negative sulfonic groups of NCC are the main driving force for anionic azo dye adsorption onto PMS and PMGS adsorbents.

## 1. Introduction

Dyes are important chemicals for many industrial activities related to textiles, paper, cosmetics, paint, etc.. However, dye residue is a kind of pollutant for the aquatic environment [1], because many dyes are highly toxic with low biodegradation [2]. Different techniques have been investigated and further developed for treatment to remove dyes from aqueous solution [3,4], such as photocatalysis [5,6,7], advanced oxidation [8,9], flocculation and coagulation [10], biological processes [11], and adsorption [12,13,14,15]. For developing countries, adsorption is one of the most suitable and effective methods for dye removal when using low-cost adsorbents such as minerals, clays or agricultural sub-products [2,16,17]. Various hybridized materials have been investigated for removal of dye through adsorption techniques, such as nanofibrous membrane [18] or porous g-C_3_N_4_ nanosheet [19]. In order to produce novel adsorbents, materials can be modified by surface coating [20] or pre-adsorption with active chemical agents [21].

Polyelectrolyte is a charged polymer in which monomer molecules contain positively or negatively charged groups [22,23]. A polycation is a polyelectrolyte with positive charge while a polyanion has a negative charge. Recent studies demonstrate that the adsorptive removal of ionic dyes in water environment is significantly enhanced when using surfactants or polyelectrolytes [2,24]. For these systems, an understanding of adsorption characteristics and mechanisms of dyes on modified adsorbents with chemical interactions are important to control and increase the removal efficiencies of organic contaminants.

Silica is the dehydrated form of silica-gel material at high temperature. Non-porous and low charge density silica and silica-gel are difficult to remove from ionic dyes directly [25,26,27]. In this case, surface modifications of silica and silica-gel are required to enhance removal efficiency of ionic dyes. Poly-diallyl-dimethylammonium chloride (PDADMAC) is a strong commercial polycation with pH independent charging behavior. PDADMAC modifies the solid surface or creates high performance adsorbents for removing some organic pollutants [28,29,30]. The adsorption of PDADMAC onto silica at different ionic strengths has also been also thoroughly investigated [31]. Nevertheless, to the best of our knowledge, the removal of anionic dye using PDADMAC modified silica and silica-gel materials has not been studied.

Synthetic azo dyes are widely used in numerous industrial activities [13,32]. Adsorption of azo dye on different systems and dye degradation have been thoroughly investigated [33,34,35,36]. However, studies on the removal of multivalent azo dyes such as new coccine (NCC) using a novel adsorbent are still inadequate. Wang et al. [37] investigated the removal of NCC using sludge particulates at different pH, suspended solid, and salt concentration. However, this group did not study the impact of the surface modification using polyelectrolyte, and adsorption isotherms were not investigated [37].

This work aims to investigate of the adsorption characteristics of NCC onto silica and silica-gel particles with surface coating by PDADMAC. The change in surface functional group of silica and silica-gel with PDADAMC before and after NCC adsorption is evaluated by Fourier transform infrared spectroscopy (FTIR). We also use different isothermal models to fit adsorption of NCC onto PDADMAC modified silica and silica-gel to suggest adsorption mechanisms.

## 2. Materials and Methods

### 2.1. Materials

**Figure 1:**
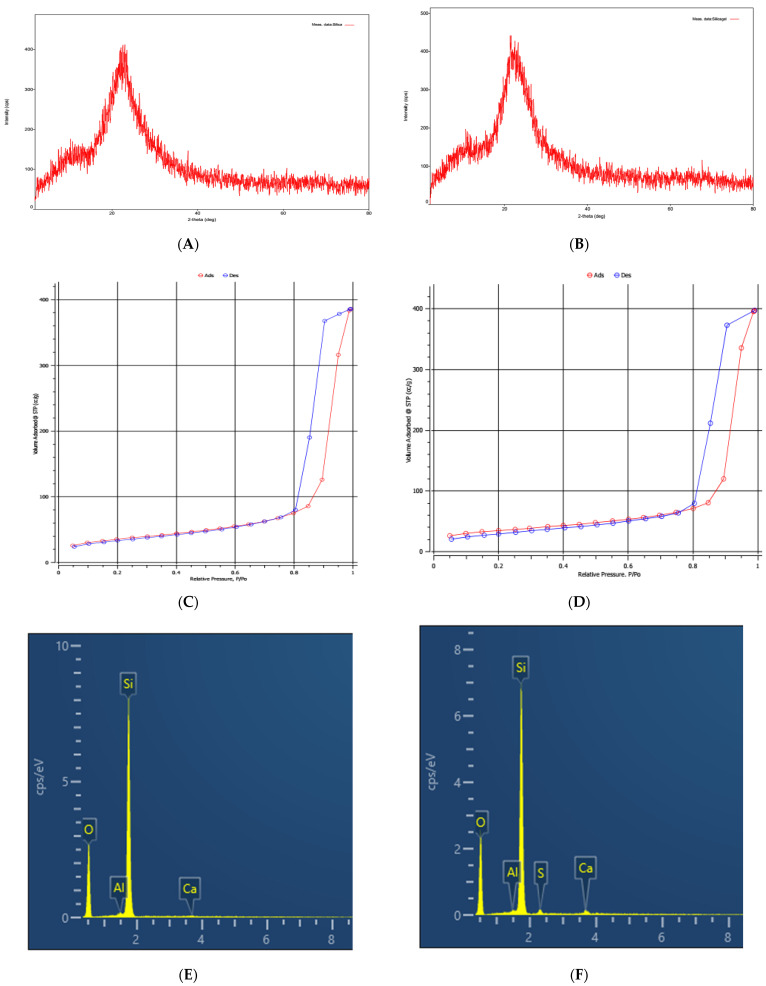
Characterization of silica and silica-gel. X-ray diffraction (XRD) patterns of silica (**A**) and silica-gel (**B**). Adsorption and desorption of N_2_ isotherms on silica (**C**) and silica-gel (**D**). The Energy Dispersive X-ray (EDX) of silica (**E**) and silica-gel (**F**).

High purity silica-gel with a diameter of about 50 μm was calcinated at 800 °C to form silica. The XRD patterns used for confirmation of phase structures of silica-gel and silica particles were conducted by an X-ray diffractometer (Bruker D8 Advance) with Cu Kα radiation (λ = 1.541862 Å). The diffraction intensity was recorded between the range of 2−80° (2θ) with a step increment of 0.02°. Figure 1 shows that both silica and silica-gel have amorphous silicon oxide with specific boarding peaks at 2theta of 20–26° [38]. The adsorption and desorption isotherms of N_2_ to calculate specific surface area according to Brunauer–Emmett–Teller (BET) theory were also conducted. The specific surface area of silica and silica-gel materials were carried out using a surface area analyzer (Nova touch 4LX, Anton Paar). Samples with a weight of about 0.12 g were measured at 77 K with liquefied N_2_ for 9 h. The specific surface areas of 159.6 and 169.7 m^2^/g were found to be for silica and silica-gel, respectively. Energy Dispersive X-ray Spectroscopy (EDX) which is used for elemental analysis of materials was conducted using Oxford Instruments. The EDX shows a high purity of silica and silica-gel with a total amount of Si and O greater than 99%.

**Figure 2:**
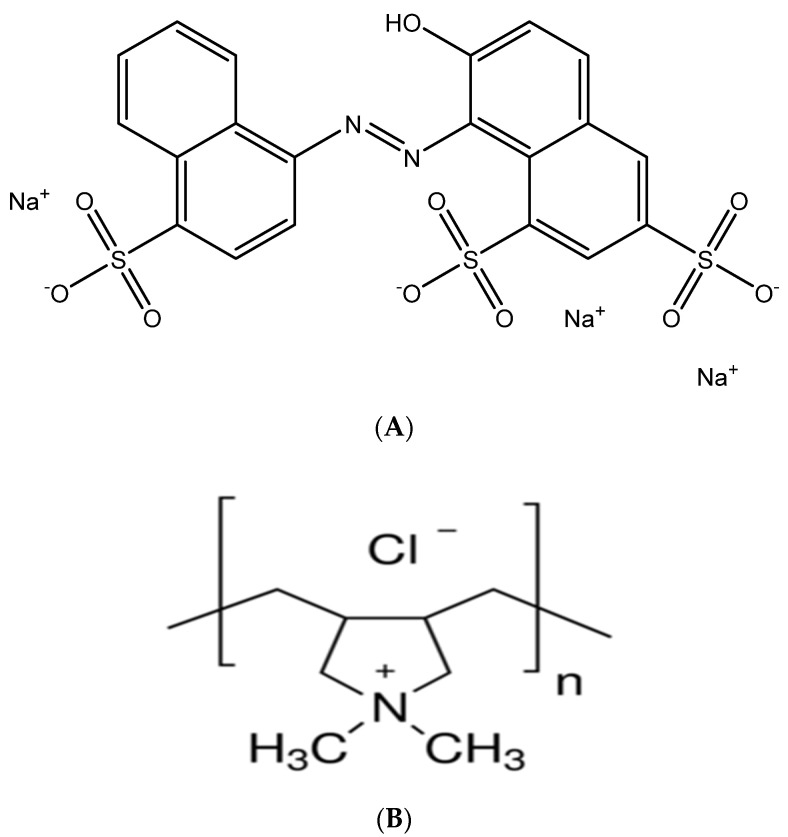
Chemical structures of anionic dye new coccine, NCC (**A**) and poly-diallyl-dimethylammonium chloride (PDADMAC) (**B**).

Anionic azo dye, new coccine (NCC), with purity higher than 85%, from Tokyo Chemical Industry (Japan) was used as an adsorbate in dye adsorption. We used a strong polycation purchased from Sigam, poly-diallyl-dimethylammonium chloride (PDADMAC) 20 wt.% in H_2_O with a molecular weight of 400–500 kg·mol^−1^. The chemical structures of NCC and PDADMAC are indicated in Figure 2. The pH and ionic strength were adjusted by KCl, HCl, and KOH. The solution pH was monitored by a HI 2215 pH meter (Hanna, Woonsocket, RI, USA). All chemicals used in the present study are analytical reagents (Merck, Germany). The ultra-pure water was produced from a Thermo Scientific Barnstead MicroPure with resistivity of 18.2 MΩ.cm.

### 2.2. Adsorption Study

Adsorption studies were conducted by batch experiments in 10 mL fancol tubes at room temperature of 22 ± 2 °C, controlled by an air conditioner. To carry out adsorption experiments, the silica and silica-gel were mixed with various initial concentrations of PDADMAC under different ionic strengths. For NCC adsorption isotherms, concentrations from 20 to 1500 mg·L^−1^ were desired and pH was adjusted to original value. The equilibrium time in dye adsorption was achieved after 60 min. The adsorption amounts of PDADMAC ($\Gamma_{\mathrm{PDADMAC}}$) onto silica and silica-gel were determined by the differences between PDADMAC concentrations before and after adsorption monitored by total organic carbon analyzer (TOC-V_CSN_). The adsorption capacities of NCC onto PDADMAC modified silica (PMS) and PDADMAC modified silica-gel (PMSG) were determined by the different concentrations of NCC solutions before adsorption and after equilibrium process by spectroscopic method.

The removal efficiency (%R) of NCC was calculated by using Equation (1).

(1) $$\mathrm{Removal}\;\left(\%R\right)=\frac{C_{i}-C_{e}}{C_{i}}\times 100\%\;$$

The adsorption isotherms of PDADMAC onto silica and silica-gel were fitted by the two-step model with general isotherm equation [39]:

(2) $$\;\Gamma =\frac{\Gamma_{\infty }k_{1}C\left(\frac{1}{n}+k_{2}C^{n-1}\right)}{1+k_{1}C\left(1+k_{2}C^{n-1}\right)}\;$$

where Γ (mg·g^−1^) and Γ_∞_ (mg·g^−1^) are the adsorbed capacity and the maximum adsorption capacity of NCC, respectively, while k_1_ (g·mg^−1^) and k_2_ (g·mg^−1^)*^n^*^−1^ are equilibrium constants for first layer of adsorption, respectively; multilayer clusters of polymer molecules are denoted by n.

Adsorption of NCC onto PMS and PMSG were fitted by Langmuir and Freundlich models.

The equation described by the Langmuir model is [40]:

(3) $$\frac{C_{e}}{q_{e}}=\frac{C_{e}}{q_{max}}+\frac{1}{q_{max}K_{L}\;}\;$$

where *C_e_* (mg·L^−1^) is the NCC equilibrium concentrations, *q_e_* (mg·g^−1^) is the NCC adsorption capacity at equilibrium time, *q_max_* (mg·g^−1^) is the maximum NCC adsorption capacity and *K_L_* (L·g^−1^) is the Langmuir constant.

T Freundlich model with equation [41] is given below:

(4) $${\mathrm{Lnq}}_{e}={\mathrm{LnK}}_{F}+\frac{1}{n}{\mathrm{LnC}}_{e}$$

where K_F_ (mg^n−1^L^n^·g^−1^) is the Freundlich constant while *n*^−1^ is the adsorption intensity.

### 2.3. Spectroscopic Method

All concentrations of NCC were quantified by spectroscopic method using an UV-Vis double-beam spectrophotometer (UV-1650 PC, Shimadzu, Japan) at a wavelength of 505 nm. The standard calibration curves at different conditions of pH and NaCl concentrations were daily conducted with a correlation coefficient of at least 0.999. All samples were directly measured, or measured after appropriate dilution by standard calibration curves.

The FT-IR spectra were conducted on JASCO, Japan (FT-IR, 4600 type A) using deuterated triglycine sulfate (DTGS) detector with a resolution of 4 cm^−1^. All solid samples were measured using KBr pellets. The wavenumber was recorded from 400 to 4000 cm^−1^.

## 3. Results and Discussion

### 3.1. Adsorption of PDADMAC onto Silica and Silicagel

**Figure 3:**
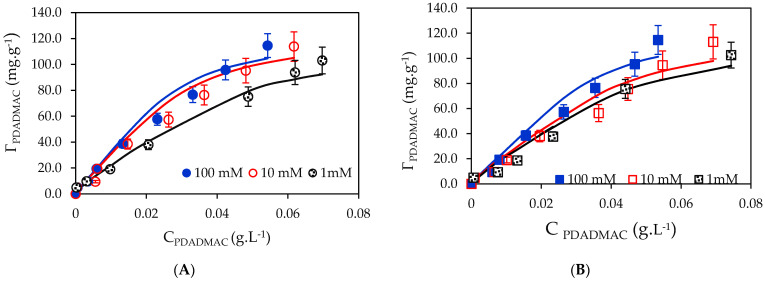
Adsorption isotherms of PDADMAC onto silica (**A**) and silica-gel (**B**) at three KCl concentrations. Points are experimental data, solid lines are the results calculated from the two-step model.

Adsorption isotherms of PDADMAC onto silica and silica-gel at three KCl concentration (pH 10) are indicated in Figure 3.

Figure 3 shows that adsorption increased with increasing KCl concentration while an increase in the number of cations K^+^ induces a decrease in the electrostatic attraction between silica, silica-gel and PDADMAC. The electrostatic attraction between the cationic PDADMA^+^ and negatively charged silica and silica-gel surfaces is decreased with an increase of KCl concentration [42]. Although the increases in PDADMAC adsorption onto both silica and silica-gel are not very high at 10 and 100 mM, the increase in adsorption capacity is clearly observed at 1 mM. This indicates that the non-electrostatic interaction plays an important role. Non-electrostatic interaction, which may be hydrophobic, lateral or hydrogen bonding, could influence the adsorption [43]. The adsorption of PDADMAC in our cases is similar to the adsorption of polyanion PSS onto alumina surface [44]. Adsorption of polyelectrolytes can eliminate the counter ions in aqueous solution, therefore adsorption may change the entropy [31,45].

Figure 3 also shows that the two-step model using the parameters in Table 1 can reasonably represent PDADMAC adsorption onto both silica and silica-gel at three ionic strengths of 1, 10 and 100 mM. Interestingly, the value of *k*_1_ did not change for all cases while the values of *k*_2_ increased with increasing KCl concentration. The different in maximum adsorption capacities for all case were insignificant, demonstrating that PDADMAC attached easily to the adsorbent with negatively charged surfaces. It also indicates that many loops and tails of PDADMAC occurred on silica and silica-gel surfaces. In addition, the values of *n* were the same with adsorbent, suggesting that PDADMAC adsorption with multilayer formation had taken place. In order to achieve a high positively charged surface, the initial PDADMAC concentration of 1 mg·mL^−1^ was selected to modify silica and silica-gel surfaces.

### 3.2. Comparison of NCC Removal Using Silica and Silica-Gel without and with PDADMAC Modification

After surface modification of silica and silica-gel with PDADMAC, the surfaces of both silica and silica-gel become highly positive charged, which can enhance the adsorption of anionic azo dye NCC [16].

**Figure 4:**
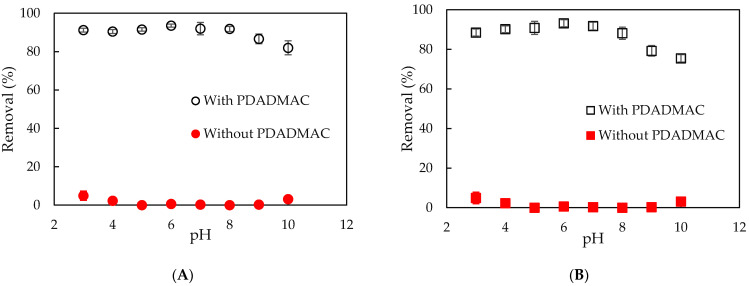
Comparison of NCC removal using silica (**A**) and silica-gel (**B**) with and without PDADMAC modification. (C_NCC_ 20 mg·L^−1^, adsorption time 30 min, adsorbent dosage 10 mg·mL^−1^). Error bars show standard deviations of three replicates.

Figure 4 shows that the NCC removal using silica and silica-gel without PDADMAC modification is extremely low at all pH in a range of 3–10 because the surface charge silica and silica-gel are completely negative at all pH values. The interaction between negatively charged NCC species with both silica and silica-gel surfaces was negligible due to the strong electrical repulsion forces. Nevertheless, the removal of NCC using silica and silica-gel increased dramatically to more than 93% after surface modification by PDADMAC (namely, PMS and PMSG adsorbents). The effect of pH for NCC removal using PMSG was more clear than that for PMS in the pH range 2–8. From pH 8–10, similar trends in significant decrease of NCC removal were observed for both PMS and PMSG because of the dissolution of silica and silica-gel [46]. The maximum NCC removal using both PMS and PMSG was reached at pH 6. Few studies have investigated NCC removal through the adsorption technique using other adsorbents. NCC removal using both PMS and PMSG has not been reported. NCC removal efficiencies using PMS and PMSG are much higher than that using silica and silica-gel without PDADMAC modification, so that further studies should only focus on PMS and PMSG adsorbents.

The optimum conditions for NCC removal using PMS and PMSG are indicated in detail below.

### 3.3. Adsorptive Removal of NCC Using PMS and PMSG

#### 3.3.1. Effect of Contact Time

**Figure 5:**
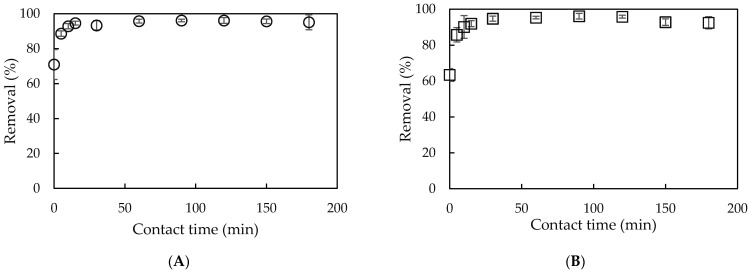
Effect of contact time on NCC removal using PMS (**A**) and PMSG (**B**). (C_NCC_ 20 mg/L, pH 6, adsorbent dosage 10 mg·mL^−1^). Error bars show the standard deviations of triplicates.

Contact time strongly induces the adsorption equilibria. The effect of contact time on the adsorptive removal of NCC using PMS and PMSG is presented in Figure 5. As can be seen, the NCC removal increased very quickly with an increase of contact time from 10 min to 60 min for both PMS and PMSG. After 60 min, the NCC removal did not change significantly. The equilibration time of NCC onto PMS and PMSG in our case is similar to NCC adsorption on sludge particulate [37]. However, the NCC removal efficiencies in this case are much higher than in the previous study. Therefore, an equilibration time of 30 min was chosen for NCC removal using PMS and PMSG.

#### 3.3.2. Effect of Adsorbent Dosage

The adsorbent dosage inducing the total surface area of adsorbent and the net surface charge [2,17] highly influence to adsorption process.

**Figure 6:**
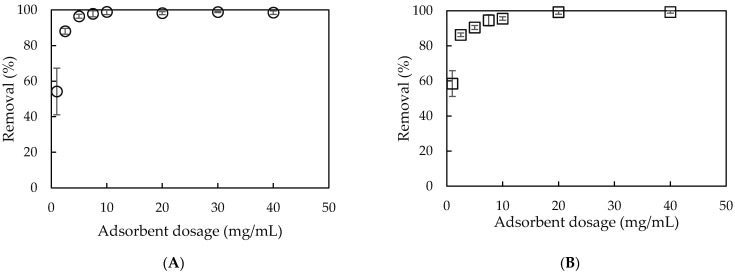
Effect of adsorbent dosage on NCC removal using PMS (**A**) and PMSG (**B**). (C_NCC_ 20 mg·L^−1^, pH 6, contact time 30 min). Error bars show the standard deviations of triplicates.

Figure 6 indicates that the removal of NCC using PMS and PMSG dramatically increased along with increasing adsorbent dosage in the range of 1–10 mg·mL^−1^ for PMS and from 1 to 20 mg·mL^−1^ for PMSG. This implies that NCC removal using PMS required less adsorbent than PMSG due to the surface activity of silica after treatment at high temperature. The increase in available binding sites and net charge density for the adsorption, with an increase in adsorbent dosage, enhanced the increase in NCC removal [47]. However, an increase in adsorbent dosage did not affect the removal due to the maximum binding sites and surface charge density. The optimum adsorbent dosages for NCC removal using PMS and PMSG were found to be 10 and 20 mg·mL^−1^, respectively.

### 3.4. Adsorption Isotherms of NCC onto PMS and PMSG

The effect of ionic strength on NCC adsorption onto oppositely charged PMS and PMSG surfaces is clearly represented by adsorption isotherms at three salt concentrations at pH 6. We used two isothermal models to fit adsorption isotherms of NCC onto PMS and PMSG. Table 2 shows the parameters of NCC adsorption isotherms onto PMS and PMSG fitted by Langmuir and Freundlich models at different KCl concentrations from 1 to 100 mM.

As can be seen in Table 2, NCC adsorption capacities decreased sharply when increasing KCl concentration from 1 to 10 mM for both PMS and PMSG, while they changed slightly from 10 to 100 mM. Adsorption of NCC on PMS and PMSG is controlled by both electrostatic and non-electrostatic interaction, but the electrostatic interaction is dominant. Maximum adsorption capacity using PMSG is always higher than that using PMS at different KCl concentrations, indicating that PMSG is better than PMS in terms of NCC removal. The Langmuir model achieved better results compared to Freundlich. This can be explained by the fact that anionic NC species may occur on the monolayer on both PMS and PMSG surfaces, although the Langmuir isotherm is based on physical adsorption [35]. In our cases, NCC adsorption onto PMS and PMSG was favored in chemical adsorption due to the presence of chemical bonding.

### 3.5. Adsorption Mechanisms of NCC onto PMS and PMSG

**Figure 7:**
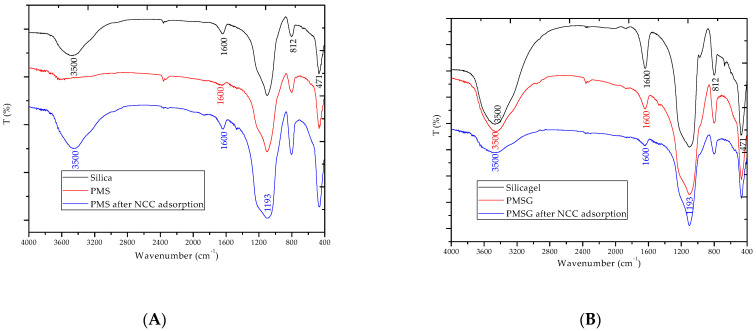
The FT-IR spectra of silica, PDADMAC modified silica (PMS) and PMS after NCC adsorption (**A**) and silica-gel, PMSG, PMSG after NCC adsorption (**B**).

We evaluated the change in surface functional group by Fourier transform infrared spectroscopy (FT-IR) in combination with adsorption isotherms to suggest adsorption of NC onto PMS and PMSG. The FT-IR spectra of silica and silica-gel with and without PDADMAC modification and after NCC adsorption are shown in Figure 7.

Figure 7 shows that silica and silica-gel after PDADMAC adsorption have similar specific peaks, except for the vibration of -OH at a wavelength of about 3500 and 1600 cm^−1^. This -OH peak appeared clearly for silica-gel because the hydroxyl group is eliminated at high temperature in the case of silica. The peaks of the -NH vibration of ammonium cations in PDADMAC at 1473.62 cm^–1^ disappeared after adsorption onto both silica and silica-gel [48]. The intensity peak at 1600 cm^−1^ for silica-gel decreased while this peak disappeared after PDADMAC modification. This suggests that hydrogen bonding may induce the adsorption of polycation onto silica and silica-gel. In addition, the peaks of 812 cm^–1^ of symmetric Si-O shifted to a shorter wavenumber while bending vibration assigned at about 471 cm^–1^ did not change after PDADMAC modification. These results indicate that PDADMAC adsorption on silica and silica-gel forms PMS and PMSG, respectively by both electrostatic and non-electrostatic interactions.

Figure 7 also shows that after NCC adsorption, the FT-IR spectra of PMS and PMSG are similar. The spectra of NC powder (data not shown) revealed that the bands at 1423, 1491, 1570 and 1632 cm^−1^ were assigned different olefin bonding of naphthalene rings or phenyl ring vibration with stretching of the C=N group, that corresponded to active groups of azo dye. These bands agree well with the spectra of NCC powder [49]. The peak at 1600 cm^−1^ assigned for -OH vibration appeared again for PMS, but its intensity still decreased for PMSG, while this peak disappeared after PDADMAC modification. We also found that the strong bands at 1193 and 1047 cm^−1^ assigned for the vibrations of the sulfonic group [32,50] of NCC disappeared after adsorption onto PMS and PMSG. The results indicate NCC adsorption via two oxygen atoms of the sulfonic group of the azo dye [32,50] and -NH groups occurring on PMS and PMSG. These are in good agreement with adsorption isotherms in which ionic strength plays a crude role due to the presence of electrostatic interaction. Based on adsorption isotherms and the change in vibration surface active groups by FT-IR, we suggest that NCC adsorption Ponto PMS and PMSG is mainly by electrostatic attraction between anionic species of NCC and positively charged PMS and PMSG surfaces.

### 3.6. Reusability of PMS and PMSG

To demonstrate the performance of adsorbents, the regeneration of adsorbent is needed to evaluate the reusability and stability of PMS and PMSG [34]. The regeneration of both PMS and PMSG were conducted using 4 M HCl. The reuse experiments were conducted in triplicate.

**Figure 8:**
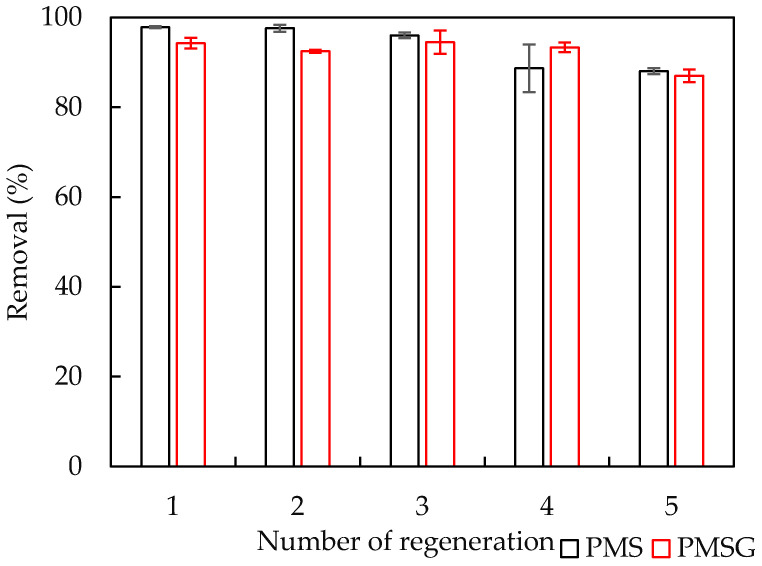
Removal of NCC using PMS and PMSG after five regenerations. Error bars show standard deviations of three replicates.

Figure 8 shows the NCC removal using PMS and after five cycles. Although the NCC removal was slightly reduced, NCC removal efficiencies were greater than 87% after five cycles when using PMS and PMSG. This implies that PMS and PMSG are reusable adsorbents with high performance for NCC removal. These materials may further apply for removal not only of dye, but also of other organic pollutants [51] with negative charges.

## 4. Conclusions

We have investigated the adsorption of anionic azo dye, NCC, onto silica and silica-gel after pre-adsorption of polycation PDADMAC. The two-step model was successfully applied to represent the experimental results of adsorption isotherms of PDADMAC onto silica and silica-gel. NCC removal using silica and silica-gel was very low, but increased dramatically when using PMS and PMSG. The maximum NCC removal using PMS and PMSG was achieved at contact time 30 min, pH 6. The optimum PMS and PMSG dosages for NCC removal were found to be 10 and 20 mg·mL^−1^, respectively. Adsorption amounts of NCC decreased with increasing salt concentration, confirming that the NC adsorption onto PMS and PMSG is mainly induced by electrostatic attraction. After five regenerations, the NCC removal efficiencies using PMS and PMSG were greater than 87%. The results of adsorption isotherms, the surface modifications by FTIR, suggested that adsorption of NCC is mainly controlled by electrostatic interaction based on the formation, between one sulfonic group on the naphthalene ring and cationic ammonium ion, of PMS and PMSG.

## Figures and Tables

**Table 1:** Fit parameters for polydiallyldimethylammonium chloride (PDADMAC) adsorption onto silica and silica-gel.

Adsorbent	C_KCl_(mM)	$\Gamma_{\infty }$(mg·g^−1^)	*k*_1_× 10^2^ (g·mg^−1^)	*k*_2_× 10^2^ (g·mg^−1^) ^n−1^	*n*
Silica	1	110	1.0	8.0	2.9
	10	120	1.0	8.0	2.8
	100	120	1.0	10	2.8
Silica-gel	1	110	1.0	8.0	3.0
	10	115	1.0	9.0	3.0
	100	120	1.0	15	3.0

**Table 2:** The parameters for NCC adsorption isotherms onto PDADMAC modified silica (PMS) and PDADMAC modified silica-gel (PMSG) at different KCl concentrations by Langmuir and Freundlich models.

Adsorbent	Model	Parameter	KCl Concentration (mM)		
			1	10	100
PMS	Langmuir	q max (mg·g^−1^)	58.08	44.64	44.92
		K_L_ (L·g^−1^)	0.029	0.099	0.065
		R^2^	0.9593	0.9985	0.9989
	Freundlich	K_F_	6.092	7.148	6.833
		*n*	2.741	3.317	3.160
		R^2^	0.6165	0.5809	0.7455
PMSG	Langmuir	q max (mg·g^−1^)	64.12	55.09	52.63
		K_L_ (L·g^−1^)	0.022	0.046	0.044
		R^2^	0.9976	0.9980	0.9814
	Freundlich	K_F_	3.282	5.879	7.804
		*n*	2.021	2.615	3.162
		R^2^	0.8201	0.6063	0.7263

## Data Availability

Not applicable.
